# Supplementary material for: Severity of non-alcoholic steatohepatitis is not linked to testosterone concentration in patients with type 2 diabetes
Source: PLoS One. 2021 Jun 2;16(6):e0251449. doi: 10.1371/journal.pone.0251449 (PMC8172043; doi:10.1371/journal.pone.0251449)
Supplement: Attachment — Submitted filename: Response_to_reviewers.docx [file pone.0251449.s002.docx]

**Kenneth Cusi, MD, FACP, FACE**	1600 SW Archer Road

Professor of Medicine	PO Box 100226

Chief, Division of Endocrinology,	Gainesville, FL 32610

Diabetes and Metabolism	Phone: 352-273-8661/7236

Fax: 352-273-7441

E-mail: [kcusi@ufl.edu](mailto:kcusi@ufl.edu)

Massimo Federici, M.D.

Academic Editor

PLOS ONE	March 26^th^, 2021

RE: PLOS ONE Resubmission of manuscript PONE-D-21-02015.

Dear Dr. Federici,

We are resubmitting for publication in *PLOS One* our revised version of the manuscript titled entitled “Severity of NASH is Not Linked to Testosterone Concentration in Patients with Type 2 Diabetes” by Dayton et al. We appreciate the kind words of support by the editors and reviewers, as well as their excellent suggestions to enhance our manuscript. We include below a point-by-point response to the issues raised. We hope that the referees and editorial board will find this improved version to meet the high standards of your prestigious Journal.

Best regards,

Kenneth Cusi, M.D., F.A.C.P., F.A.C.E.

Professor of Medicine

Response to referee

**Referee #1:** **I read with interest the manuscript of Dayton et al regarding the influence of liver disease on testosterone in males withT2DM. In this cross-sectional study of 175 males with T2DM, NAFLD was linked to lower total testosterone in patients with T2DM, but likely given a common soil of insulin resistance/obesity and not from the severity of liver necroinflammation or fibrosis.**

Thank you for your support.

**The manuscript is interesting and well written. I have some points:**

**Q1. In figure 1, could you perform also a test to evaluate intra-group differences?**

After adjustment for multiple comparisons, between-group comparisons in Figure 1 are:

Group 1 vs. 2: p = 0.19

Group 2 vs. 3: p = 0.99

Group 1 vs. 3: p = 0.13

(The p we used was a p for trend so it makes sense that comparisons between groups are NS)

Data included in the legend (page 10).

**Q2. In figure 4, could you perform also a test to evaluate intra-group differences for panel A and D?**

Fig 4 A. After adjustment for multiple comparisons, between-group comparisons are:

Steatosis grade 1 vs. 2: p = 0.003

Steatosis grade 2 vs. 3: p = 0.99

Steatosis grade 1 vs. 3: p = 0.37

Fig4 D. After adjustment for multiple comparisons, between-stage groups comparisons are:

Stage 0 vs. 1: 0.56

Stage 1 vs. 2: 0.99

Stage 0 vs. 2: 0.65

Stage 0 vs. 3: 0.99

Stage 2 vs. 3: 0.99

Data included in the legend (page 12).

**Q3. In Figure 3, were the variables logarithmically transformed? Based on Table 1, they seem not normally distributed.**

We appreciate this important question from the reviewer. In Figure 3 variables were not logarithmically transformed because only liver fat and HOMA were not normally distributed based on Shapiro-Wilk normality tests. Testing these variables after logarithmic transformation, produced similar correlations to those reported in Fig. 3:

Total testosterone and liver fat: -0.28, p=0.002.

Total testosterone and HOMA: -0.30, p<0.001.

Due to the fact that the relationships were similar, for simplicity of interpretation for the readers, we decided to present the data without log transformation.

**Q4. In page 12 the authors wrote: “In order to assess whether the association between total plasma testosterone and intrahepatic triglyceride content was independent of other metabolic factors, we performed a multiple linear regression analysis. Intrahepatic triglyceride content was independently associated with total testosterone levels (ß=-4.8, p=0.004). Body mass index (ß=-9.6, p<0.001) and HbA1c (ß=-19.8, p=0.02) were also independently associated with total testosterone in the multiple linear regression”. Please, could you report a table with these data?**

This is a great suggestion and will add to the scientific merit of the paper. The table (below) was created and added to the manuscript as well.

|  | Coefficient | p | 95% confidence interval | |
| --- | --- | --- | --- | --- |
| Liver fat content | -4.8 | 0.004 | -7.9 | -1.6 |
| HbA1c | -19.8 | 0.024 | -37.0 | -2.7 |
| BMI | -9.6 | <0.001 | -13.8 | -5.3 |
| ALT | 0.8 | 0.016 | 0.2 | 1.5 |

Table 2: Results of multiple linear regression analysis for variables associated with total testosterone. HDL, TG, Age, FPG, AST were also tested but were not independently associated with total testosterone.
